# Local Inflammatory Response after Intramuscularly Implantation of Anti-Adhesive Plasma-Fluorocarbon-Polymer Coated Ti6AI4V Discs in Rats

**DOI:** 10.3390/polym13162684

**Published:** 2021-08-11

**Authors:** Charlotte Koppe, Andreas Hoene, Uwe Walschus, Birgit Finke, Holger Testrich, Christopher Pohl, Nico Brandt, Maciej Patrzyk, Jürgen Meichsner, Barbara Nebe, Michael Schlosser

**Affiliations:** 1 Department of General Surgery, Visceral, Thoracic and Vascular Surgery, University Medical Center Greifswald, 17487 Greifswald, Germany; charlotte.koppe@stud.uni-greifswald.de (C.K.); hoene@uni-greifswald.de (A.H.); walschus@web.de (U.W.); christopher.pohl@uni-greifswald.de (C.P.); nico.brandt@protonmail.com (N.B.); patrzyk@uni-greifswald.de (M.P.); 2 Leibniz Institute for Plasma Science and Technology (INP), 17487 Greifswald, Germany; finke-hgw@t-online.de (B.F.); holger.testrich@inp-greifswald.de (H.T.); 3 Institute of Physics, University of Greifswald, 17487 Greifswald, Germany; meichsner@physik.uni-greifswald.de; 4 Department of Cell Biology, University Medical Center Rostock, 18057 Rostock, Germany; barbara.nebe@med.uni-rostock.de

**Keywords:** titanium (Ti) alloys, low-temperature plasma polymerization, plasma-fluorocarbon-polymer, anti-adhesive surface, inflammatory/immunological response, intramuscularly implantation

## Abstract

Orthopaedic implants and temporary osteosynthesis devices are commonly based on Titanium (Ti). For short-term devices, cell-material contact should be restricted for easy removal after bone healing. This could be achieved with anti-adhesive plasma-fluorocarbon-polymer (PFP) films created by low-temperature plasma processes. Two different PFP thin film deposition techniques, microwave (MW) and radiofrequency (RF) discharge plasma, were applied to receive smooth, hydrophobic surfaces with octafluoropropane (C_3_F_8_) or hexafluorohexane (C_6_F_6_) as precursors. This study aimed at examining the immunological local tissue reactions after simultaneous intramuscular implantation of four different Ti samples, designated as MW-C_3_F_8_, MW-C_6_F_6_, RF-C_3_F_8_ and Ti-controls, in rats. A differentiated morphometric evaluation of the inflammatory reaction was conducted by immunohistochemical staining of CD68+ macrophages, CD163+ macrophages, MHC class II-positive cells, T lymphocytes, CD25+ regulatory T lymphocytes, NK cells and nestin-positive cells in cryosections of surrounding peri-implant tissue. Tissue samples were obtained on days 7, 14 and 56 for investigating the acute and chronical inflammation (n = 8 rats/group). Implants with a radiofrequency discharge plasma (RF-C_3_F_8_) coating exhibited a favorable short- and long-term immune/inflammatory response comparable to Ti-controls. This was also demonstrated by the significant decrease in pro-inflammatory CD68+ macrophages, possibly downregulated by significantly increasing regulatory T lymphocytes.

## 1. Introduction

Titanium alloys are still among the most commonly used materials for metallic implants in orthopedic and trauma surgery for long-term use such as joint or hard tissue replacement as well as in temporary fracture fixation devices, including internal and external fixators, intra-medullary nails and screws. This is mainly due to their excellent biocompatibility, high corrosion resistance and low ion-formation tendency [1], which leads to encouraged cell adhesion and the osseointegration process. While this is of great importance for long-term prosthetic applications, the requirements for short-term implants are quite different. The purpose of temporary implants is to stabilize the fragments of fractured bone in order to allow healing and repair processes to occur but not to integrate into the bone to ensure safe removal [2]. However, to the present day the retrieval of metallic fracture-fixation devices is still associated with a risk of nerve and soft tissue damage, and the possibility of harming the newly formed bone by using a great amount of pullout force, and longer operation times [3,4].

After immediate surface adsorption of blood proteins, the interaction of cells and the implant as well as the characteristics of the metallic surface have a great influence on the initial attachment process. This is mediated by integrin receptors, leading to adhesion as well as a primary immune response of the body [5,6]. It is well established in the literature that the surface microtopography such as roughness or micro-discontinuities, hydrophilicity/-phobicity, chemistry and charge influence molecular adhesion processes [7,8,9]. Hayes et al. demonstrated that reduced surface roughness through polishing leads to a significant lower osteointegration in vivo [2].

In recent years, low-pressure and low-temperature plasma processes, as a technique for metal surface modification through thin film deposition, have become more important in biomedical engineering. One of the reasons is that the coating can be highly specific, while the characteristics of the coated metal itself remain unchanged resulting in extremely effective implant performance [10].

Considering the initial assumption that the surface structure and charge is a crucial factor for tissue integration, the influence of plasma coating has been demonstrated in previous studies which showed that titanium platelets coated with a thin positively charged plasma polymer from allylamine (PPAAm) had a beneficial effect on osteoblastic adhesion (MG63 cells) in vitro [11] and, dependent on the chosen plasma process conditions, caused an increased or decreased local immune response compared to uncoated controls following implantation in vivo [12].

In contrast, anti-adhesive coatings are beneficial for temporary devices by inhibiting cell attachment, colonisation and growth on the titanium surface. The modification of titanium surfaces by low-temperature plasma thin film deposition is a very promising method for improvement of implant performance. Concerning thin film deposition for anti-adhesive coatings, the use of plasma-fluorocarbon-polymer (PFP) precursors have several advantages including easy thickness control, excellent adhesion to the coated metal and the ability to create a hydrophobic surface [13]. Hydrophobic surfaces tend to have a higher water contact angle than hydrophilic surfaces leading to a reduced wettability, effecting cell attachment and proliferation as described to be higher on hydrophilic than on hydrophobic surfaces [14,15,16]. The host immune response is determined by the interaction between the implanted material surface and surrounding tissue. Clinically a pro-inflammatory immune response is directly associated with prolonged reconstruction processes and complicated delayed wound healing [17,18].

In general, the immune response after tissue injury due to biomaterial implantation changes in a time-dependent manner. Innate immune cells such as neutrophils recognize danger associated molecular patterns (DAMPs) [19]. Through cytokines like IFN-γ, they coordinate the recruitment of circulating monocytes and initiate the differentiation into different phenotypes of macrophages according to the occurring environment of pro- or anti-inflammatory chemical signals. This is determined by a complex multifactual influence and is also affected by the health condition of the host tissue [20]. Within a few hours following implantation, monocytes/macrophages as early responders, become the dominant cell type in the surrounding tissue [21].

In response to INF-γ, monocytes progress into pro-inflammatory M1-type macrophages. These cells are the key actors in initiating a TH1-response, characterized by INF-γ as the predominant cytokine, which causes a pro-inflammatory response. Although this is important to initiate the natural wound healing process, it can lead to tissue damage in cases of prolonged activation. M1-type macrophages, identified by expression of markers such as CD68 and CD80 [6], can differentiate into M2-type tissue macrophages, known for their anti-inflammatory modulation. Among other markers, M2 macrophages express CD163. Due to their ability of tissue remodeling and repair, this macrophage switch is essential for the tissue healing and integration processes. However prolonged or overactivation may cause fibrous encapsulation of the implanted devices, impeding a successful implantation. As common in immune responses both phenotypes are crucial for successful tissue healing and remodeling, but balance and sequence of activation are essential [20].

Other important innate immune cells are natural killer cells (NK cells). Apart from their more widely known functions as cytotoxic and cytokine producing cells, NK cells also seem to be engaged in the regulation of other immune cells such as dendritic cells or T lymphocytes. This highlights the possibility of NK cells having the ability to affect the immune response towards a more anti-inflammatory or pro-inflammatory microenvironment [22].

Consistent with the inflammatory reaction described above, professional antigen-presenting cells (MHC class II antigen-presenting cells) are responsible for initiating the adaptive immune answer. Only dendritic cells, macrophages and B cells express MHC class II antigens and are therefore able to present antigens to the T cell-mediated branch of humoral immune response and activate effector T cells [23]. Concerning T cell activation, Interleukin-2 (IL-2) receptor-positive regulatory T cells are of great interest. IL-2 is a cytokine with a pleiotropic biological mechanism reaching from immunostimulatory effects via cytotoxic CD8+ cell activation to immunosuppressive effects by stimulating CD4+ regulatory T cells (T_reg_) which are important for inducing an anti-inflammatory environment characterized by high levels of IL-10 and TGF-β [24].

While the inflammatory reaction has a great influence on tissue healing, muscle regeneration and vascularization contribute significantly to this process. Myofiber regeneration is a complex process similar to muscle formation during embryogenic development. Intermediate filament (IF) proteins are a necessary component of this pathway. Concerning muscle injury, the IF-protein nestin is of great interest since it has been observed that its upregulation is essential for the induction of myogenic differentiation [25,26].

The aim of the present study was an immunological in vivo evaluation of different anti-adhesive PFP-films on titanium alloy samples, investigating their influence on the local inflammatory tissue response after implantation in Lewis rats. In previous studies Finke et al. examined and characterized titanium alloy platelets (Ti6AI4V) which were coated with different PFP-films precursors and different discharged plasma either applied by means of microwave (MW: higher electron densities and lower electron energies) or radiofrequency (RF: ion bombardments on growing film). This process resulted in very smooth, abrasion resistant, cross-linked PFP-films designated as MW-C_3_F_8_, MW-C_6_F_6_ and RF-C_3_F_8_. All implants exhibited the desired thin film with anti-adhesive properties and a stable hydrophobic character. In vitro studies revealed a decrease in the number and size of MG-63 (Osteoblast) cells cultivated on those PFP-modified titanium samples in comparison to attachment on a non-coated control implant, emphasizing the anti-adhesive character of PFP [27].

The present study postulates that an anti-adhesive coating of titanium implants also evokes a decreased inflammatory local reaction in vivo. To examine this hypothesis, a simultaneous intramuscular implantation of the four different samples (control, RF-C_3_F_8_, MW-C_3_F_8_ and MW-C_6_F_6_) was performed in an established rat animal model for immunohistochemical examination of calcium phosphate-coated titanium implants [28]. For this, a differentiated morphometric evaluation of the inflammatory reaction in the surrounding peri-implant tissue over time (acute phase day 7, intermediate phase day 14 and chronic inflammation day 56) was conducted by immunohistochemical staining of CD68+ monocytes/macrophages (ED1), CD163+ macrophages (ED2), MHC class II positive antigen-presenting cells (OX6), T lymphocytes (R73), CD25+ (IL-2R+) regulatory T lymphocytes (OX39), activated NK cells (ANK61) and nestin-positive cells (Rat 401).

## 2. Materials and Methods

### 2.1. Implants and PFP Film Preparation

Titanium alloy plates (Ti6AI4V) with the measurements of 5 × 5 × 1 mm and a defined roughness of Ra = 0.28 µm (Ti6Al4V-P cp, DOT GmbH, Rostock, Germany) were polished and used for low temperature plasma modifications.

The platelets were surface-modified with a plasma-fluorocarbon-polymer (PFP) film using octafluoropropane (C_3_F_8_) or hexafluorohexane (C_6_F_6_) as a precursor (Linde Gas AG, Munich, Germany) with a purity of 99.95%, and added H_2_ (Messer-Griesheim GmbH, Ludwigshafen, Germany) with a purity of 99.9999%, in the specific manner that the procedure of either microwave- (MW) or capacitively coupled radio frequency (RF)-generated low-temperature plasma discharge demands.

In previous studies optimized parameters for the PFP-film were investigated for minimum cell adhesion and found to be as follows for PFP-MW: 500 W, 10–30 Pa, 1000 s//(2.45 GHz, 500–1200 W, 10–50 Pa, 300–1000 s) and for PFP-RF: 25 W, 20 Pa, 400 s//(13.56 MHz, 20–150 W, 20 60 Pa, 20–1000 s) [27].

The two discs that were treated with a microwave coating process were placed 9 cm downstream of the MW coupling window in an industrial low-plasma reactor (V55G, Plasma finish, Germany). One alloy platelet was steamed with a C_3_F_8_-precursor and H_2_ mixture (C_3_F_8_/H_2_) and the other one with a C_6_F_6_-precursor and H_2_ mixture (C_6_F_6_/H_2_).

The radiofrequency processed samples were placed in a stainless-steel vacuum chamber of 400 mm in diameter and height, on top of a planar electrode which was powered by an RF-generator through a matching network. In order to guarantee low-germ processing environments both reactors used for deposition were combined with a laminar flow box.

Sample series were designated as MW-C_3_F_8_, MW-C_6_F_6_ and RF-C_3_F_8_, respectively. The titanium alloy platelets serving as control plates were without a PFP-film preparation.

### 2.2. In Vivo Investigations

Twenty-four male Lewis rats (age 100 days) were used in this study. All animal experiments and maintenance were approved by the LALLF Mecklenburg-West Pomerania (reference number 7221.3-1.1-074/11) and performed in accordance with the animal protection law of the Federal Republic of Germany (version of 1 January 1987), which determinates the principles of care for animals in laboratories (proposed by the National Society for Medical Research) and followed the Guideline for Keeping and Using Laboratory Animals (NIH Publication No. 80-23, revised 1985).

Each animal received four implants, one control plate and one sample from each PFP series (RF-C_3_F_8_, MW-C_3_F_8_, MW-C_6_F_6_). As established in previous studies, this approach was chosen to reduce the level of variability among the experimental animals through intra-individual comparison of the inflammatory reactions against samples vs. its own control [12,28]. Accordingly, the samples were simultaneously implanted into small intramuscular pockets (i.m.) clockwise, starting with the control sample in the upper left area of the neck muscle. The intramuscular pockets were separated by at least 2 cm from each other to avoid overlapping local inflammation effects. The implants were fixed with a non-resorbable synthetic polypropylene suture (PROLENE, Ethicon Endo-Surgery, Inc., Hamburg, Germany) to ensure relocation and to track samples on explanation dates. For seven days after the procedure, the rats were screened daily for any untypical behavior, signs of pain and severe weight loss according to a set scoring sheet.

On days 7, 14 and 56 after implantation, eight randomly selected animals were euthanised by carbon dioxide inhalation and the samples with the surrounding peri-implant tissue were carefully explanted. Using the laboratory freezer spray New Envi-Ro-Tech (Thermo Electron Corporation, Pittsburgh, PA, USA), the samples were frozen instantaneously and the Ti6AI4V platelets were carefully removed. The resulting pockets were filled with Shandon Cryomatrix embedding medium (Thermo Electron Corporation, Pittsburgh, PA, USA). For later histological examination, the tissue samples were shock frozen in liquid nitrogen and stored at −80 °C.

### 2.3. Histological Examination

Cryosections with a thickness of five µm were generated from each tissue sample using a 2800 Frigocut N cryotome (Reichert-Jung, Nussloch, Germany). Seven different antibody-based immunohistochemical staining methods were carried out to detect CD68+ monocytes/macrophages (ED1), CD163+ macrophages (ED2), MHC class II positive antigen-presenting cells (OX6), T lymphocytes (R73), CD25+ (IL-2R+) regulatory T lymphocytes (OX39), activated NK cells (ANK61) and nestin-positive cells (Rat 401). Each staining procedure was conducted according to the manufacturer’s protocol (MorphoSys AbD Serotec GmbH, Düsseldorf, Germany). The specific antibody-stained cells were visualized using the APAAP detection system (mouse monoclonal antibody alkaline-phosphatase-anti-alkaline-phosphatase; APAAP, clone AP1B9, Sigma-Aldrich Chemie GmbH, Munich, Germany) and the polyclonal rabbit anti-mouse-immunoglobulin (Z259, Dako DenmarkA/S, Glostrup, Denmark) using New Fuchsin as chromogen. Additionally, mast cells were detected by histochemical toluidine blue staining. Using a digital camera DP20 on a light microscope CX41 (Olympus, Hamburg, Germany), histological images of the different stained sections were obtained.

### 2.4. Morphometry

The obtained images were morphometrically evaluated by counting of positively stained cells in defined areas using the digital image analysis software ImageJ v1.41 (US National Institutes of Health, Bethesda, Rockville, MD, USA). Five representative squares per histological section of 20,000 pixels along the border zone between surrounding tissue and implant-free pocket were selected for detailed analysis, resulting in a total analyzed area of 100,000 pixels per image. In the chosen microscopic magnification of 100×, one pixel equals an area of 0.4796 µm^2^. The results were expressed either as a percentage of positively stained areas (for ED1, ED2, OX6, nestin) or cells per mm^2^ for markers with low cell numbers (for R73, OX39, toluidine blue, ANK61).

### 2.5. Statistics

Data are given as median and interquartile range. All tests were performed two-tailed with *p*-values of less than 0.05 considered as statistically significant. For comparison of measured percentages of positively stained area or positive cells per mm^2^ in the peri-implant tissue of implant samples, the non-parametric Kruskal–Wallis test with Gaussian approximation was used. For comparison of implant samples between the different experimental days, the Kruskal–Wallis test with Dunn’s multiple comparison post-hoc-test was used for non-paired data sets. For comparison between different implants and different cellular markers on each experimental day, the Mann–Whitney U-test was used. All statistical analysis was performed using the software GraphPad Prism version 4.02 (GraphPad Software, Inc., San Diego, CA, USA).

## 3. Results

### 3.1. Overview of Time Course of Different Inflammatory Cell Populations

After simultaneous implantation of different anti-adhesive PFP-coated and uncoated control titanium alloy plates (Ti6AI4V), a distinct time course pattern of inflammatory investigated cells could be observed (Table 1). Whereas the positively stained area of CD68+ monocytes and macrophages (ED1) significantly decreased from day 7 to day 56 for all implants, the positively stained area of CD163+ macrophages (ED2) did not change. Additionally, for MHC class II antigen-presenting cells (OX6), a significant decrease in the positively stained area could be observed for all implants. Except for the RF-C_3_F_8_ implants, this was also seen for the nestin-positive cells. Whereas the number of total T lymphocytes (R73) did not change, the number of IL-2R+ regulatory T lymphocytes (OX39) significantly increased from experimental day 7 to 56. The number of mast cells was constant for three implants, whereas for the MW-C_6_F_6_ implants a significant change was observed. Moreover, for NK cells (ANK61), the number of positive cells significantly changed over the examined period.

### 3.2. Morphometric Analysis of Different Inflammatory Cell Population

#### 3.2.1. CD68+ Monocytes and Macrophages (ED1)

**Figure 1:**
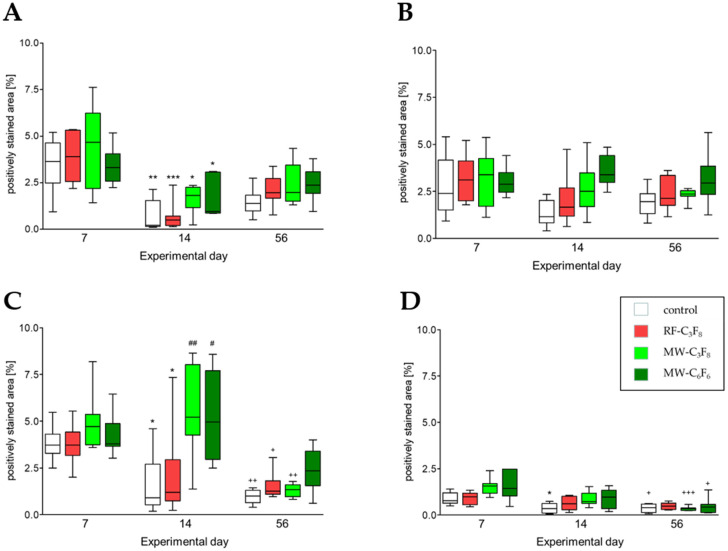
(**A**–**D**). Quantity of positively stained area of (**A**) pro-inflammatory CD68+ (ED1) monocytes/macrophages and (**B**) anti-inflammatory CD163+ (ED2) macrophages, (**C**) MHC class II+ antigen-presenting cells (OX6) and (**D**) nestin-positive cells (Rat 401) in the peri-implant tissue surrounding uncoated Ti6AI4V plates (white bars) and Ti6AI4V plates coated with the anti-adhesive plasma-fluorocarbon-polymers RF-C_3_F_8_ (red bars), MW-C_3_F_8_ (light green bars) and MW-C_6_F_6_ (dark green bars) at days 7, 14 and 56 after simultaneous intramuscular implantation in rats. Boxes represent the median and interquartile range of eight rats per experimental day; *p*-values represent significant differences between experimental days. * *p* < 0.05, ** *p* < 0.01, *** *p* < 0.001 (day 7 vs. day 14), ^+^ *p* < 0.05, ^++^ *p* < 0.01, ^+++^ *p* < 0.001 (day 7 vs. day 56), # *p* < 0.05, ## *p* < 0.01 (day 14 vs. day 56), Kruskal–Wallis test with Dunn’s post-hoc test.

The time course of pro-inflammatory CD68+ monocytes and macrophages (ED1) is demonstrated in Figure 1A. The positively stained area significantly decreased from day 7 to day 14 for control implants (*p* < 0.01) and more pronouncedly for RF-C_3_F_8_ implants (*p* < 0.001). Additionally, for both MW implant series, the positively stained area significantly decreased (*p* < 0.05). The median positively stained area varied for all implants between median 3.32–4.66% (interquartile range (IQR) of 2.2–6.23%). On day 14 it fluctuated between median 0.215–1.81% (IQR 0.15–3.07%), and on day 56 between median 1.385% to 2.36% (IQR 0.98–3.44%) for the four different implants. In comparison to control implants, a significantly higher percentage of positively stained area in the surrounding tissue of MW-C_3_F_8_ discs could be observed on day 14 (*p* = 0.0281) and for MW-C_6_F_6_ compared to RF-C_3_F_8_ implants on day 56 (*p* = 0.0111) A similar trend of increase was also seen for MW-C_6_F_6_ platelets on day 14. In contrast and compared to control implants, the RF-C_3_F_8_ discs did not induce a significantly different local inflammatory response on any experimental day.

#### 3.2.2. CD163+ Macrophages (ED2)

Figure 1B shows the time course of anti-inflammatory CD163+ macrophages (ED2). Overall, the CD163+ cells did not show any change between the three experimental time points. No switch to an anti-inflammatory macrophage population, which is essential for the tissue healing and integration processes, could be observed. The positively stained area, representing the percentage of tissue macrophages in the peri-implant tissue, remained constant, reflected in similar medians on all observation days. On day 7 the median for all implants varied around the median of 2.38–3.38% (IQR 1.51–4.24%). On day 14 it fluctuated between 1.15% and 3.39% (IQR 0.85–4.44%). On day 56 it ranged from 1.96–2.94% (IQR 1.31–2.38%). In accordance with the CD68+ monocytes and macrophages, the RF-C_3_F_8_ implants showed a similar attraction of CD163+ tissue macrophages as controls throughout the examined time course. The invasion of tissue macrophages was significantly stronger for MW-C_3_F_8_ (*p* = 0.0379) and more pronounced for MW-C_6_F_6_ (*p* = 0.0003) two weeks after implantation compared to controls. No significant difference between the four implants was found on day 7 and day 56.

#### 3.2.3. MHC Class II Antigen-Presenting Cells (OX6)

Figure 1C shows the time course of positively stained area for MHC class II positive cells, a surface structure that is essential for antigen presentation and is responsible for initiating the adaptive immune response, throughout the examined study period. A significant decrease was seen between days 7 and 14 for control and RF-C_3_F_8_ plates (*p* < 0.05), between days 7 and 56 for RF-C_3_F_8_ (*p* < 0.05), control and MW-C_3_F_8_ plates (*p* < 0.01), and between days 14 and 56 for MW-C_3_F_8_ and MW-C_6_F_6_ plates. The positively stained area, resembling the percentage of MHC class II positive cells in the peri-implant tissue, varied on day 7 for all implants, around the medians of 3.71% to 4.71% (IQR 3.17–5.36%). On day 14 it fluctuated between 0.9% and 4.9% (IQR 0.53–7.7%), and on day 56 it drastically reduced compared to day 14 with a smaller range from 1% to 2.34% (IQR 0.64–3.39%). No significant difference among the four implants was observed on day 7. The MHC class II positive stained area differs significantly for MW-C_3_F_8_ (*p* = 0.037) and for MW-C_6_F_6_ (*p* = 0.02) from control discs as well as between RF-C_3_F_8_ and MW-C_3_F_8_ (*p* = 0.014) and RF-C_3_F_8_ and MW-C_6_F_6_ implants (*p* = 0.0426) on day 14. On experimental day 56 the MW-C_6_F_6_ showed a significantly stronger reaction when compared to both the controls (*p* = 0.0047) and the MW-C_3_F_8_ implants (*p* = 0.0379).

#### 3.2.4. Nestin-Positive Cells/Area

In general, excluding the RF-C_3_F_8_ discs, a significant reduction in nestin-positive stained area in the peri-implant tissue was seen for all samples until day 56 (Table 1, Figure 1D). Nestin is essential for the induction of myogenic differentiation and its upregulation can be associated with ongoing tissue healing processes. A significant decrease was seen between days 7 and 14 for control discs (*p* < 0.05) and between days 7 and 56 for control (*p* < 0.05), MW-C_3_F_8_ (*p* < 0.001) and MW-C_6_F_6_ plates (*p* < 0.05). The percentage of nestin-positive area varied on day 7 for all implants between medians of 0.77 to 1.56% (IQR 0.56–2.48%). On day 14, it decreased and fluctuated between 0.34 and 0.97% (IQR 0.11–1.33%) and on day 56 the medians ranged from 0.33% to 0.48% (IQR 0.13–0.64%). In contrast to all the other examined markers, nestin showed a significantly stronger reaction for MW-C_3_F_8_ compared to both controls (*p* = 0.014) and RF-C_3_F_8_ implants seven days after implantation. Furthermore, on day 14 the positive area significantly increased for MW-C_3_F_8_ compared to control implants (*p* = 0.014).

#### 3.2.5. T Lymphocytes (R73)

**Figure 2:**
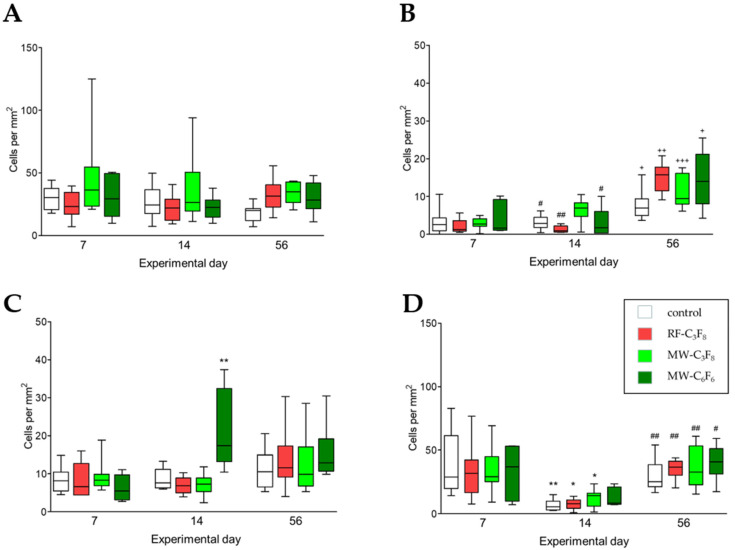
(**A**–**D**). Quantity of positively stained cells per mm^2^ for (**A**) total T lymphocytes (R73), (**B**) IL-2R+ regulatory T lymphocytes (OX39), (**C**) mast cells (toluidine blue staining) and (**D**) activated NK cells (ANK61) in the peri-implant tissue surrounding uncoated Ti6AI4V plates (white bars) and Ti6AI4V plates coated with the anti-adhesive plasma-fluorocarbon-polymers RF-C_3_F_8_ (red bars), MW-C_3_F_8_ (light green bars) and MW-C_6_F_6_ (dark green bars) at days 7, 14 and 56 after simultaneous intramuscular implantation in rats. Boxes represent the median and interquartile range of eight rats per experimental day; *p*-values represent significant differences between experimental days. * *p* < 0.05, ** *p* < 0.01 (day 7 vs. day 14), ^+^ *p* < 0.05, ^++^ *p* < 0.01, ^+++^ *p* < 0.001 (day 7 vs. day 56), # *p* < 0.05, ## *p* < 0.01 (day 14 vs. day 56), Kruskal–Wallis test with Dunn’s post hoc test.

Overall, as demonstrated in Figure 2A, the number of counted T-lymphocytes, cells of the adaptive immune system, did not show any significant change during the observation period. Due to low cell numbers the counted amount was expressed as cells per mm^2^. On day 7 the median count varied for all implants around 23–36 cells per mm^2^ (IQR 15–55 cells). On day 14 it fluctuated between 22 and 26 cells per mm^2^ (IQR 12–51 cells), and on day 56 the median ranged from 20 to 35 cells per mm^2^ (IQR 12–43 cells). The highest number of cells compared to controls was counted on day 56 for all three PFP series samples, a significant increase was observed for RF-C_3_F_8_ (*p* = 0.0281) and MW-C_3_F_8_ discs (*p* = 0.007). No difference between the four implants was seen on days 7 and 14.

#### 3.2.6. IL-2R+ Regulatory T Lymphocytes (OX39)

In general, and in comparison with T lymphocytes, the number of IL-2R+ regulatory T lymphocytes was low. T_reg_ are important cells in inducing an anti-inflammatory environment. In contrast to the R73+ T-lymphocytes and all other markers investigated, the IL-2R+ regulatory T lymphocytes showed a significant increase in cells per mm^2^ over time, reaching their highest numbers for all four implants on day 56 (Table 1, Figure 2B). In particular, a significant increase was seen between days 7 and 56 for all four implants (*p* < 0.05–*p* < 0.001), and between days 14 and 56 for control discs (*p* < 0.05), RF-C_3_F_8_ (*p* < 0.01) as well as MW-C_6_F_6_ plates (*p* < 0.05). The increase in cell number per area was accompanied by a constantly increasing median. On day 7, the median count varied for all implants around 1 to 3 cells per mm^2^ (IQR 1–9 cells). On day 14 it fluctuated between one and seven cells per mm^2^ (IQR 0–8 cells). On day 56 the median increased again, ranging from 7 to 16 cells per mm^2^ (IQR 5–21 cells). On day 14 the number of positive cells was significantly increased for MW-C_3_F_8_ in comparison with both control (*p* = 0.0281) and RF-C_3_F_8_ implants (*p* = 0.0127), whereas on day 56 the number of positive cells was significantly higher for RF-C_3_F_8_ implants compared to controls (*p* = 0.003).

#### 3.2.7. Mast Cells

Figure 2C shows the time course of mast cells stained with toluidine blue. Mast cells can ameliorate an anti-inflammatory immune response. Overall, except for MW-C_6_F_6_ discs, there was no significant change for any of the other coated Ti6AI4V implants in the present study. In comparison to day 7, the number of mast cells was significantly increased on day 14 for the MW-C_6_F_6_ discs (*p* < 0.01). On day 7 the median count of cells per mm^2^ varied for all implants around six to eight cells per mm^2^ (IQR 3–13 cells). On day 14 the median for all implants fluctuates between 7 and 17 cells per mm^2^ (IQR 5–32 cells). On day 56 the median ranged from 10 to 13 cells per mm^2^ (IQR 7–19 cells). Particularly on day 14 for the MW-C_6_F_6_ implants, the number of mast cells was significantly increased compared to their respective controls (*p* = 0.0037), RF-C_3_F_8_ (*p* = 0.0003), MW-C_3_F_8_ implants (*p* = 0.0006), whereas the number of cells between different implants did not differ on days 7 and 56.

#### 3.2.8. Activated NK Cells

Figure 2D demonstrates a significant change in the number of natural killer cells (NK) in the peri-implant tissue of all samples throughout the examined study period, with a pronounced decrease at day 14 and a subsequent increase until day 56. The number of positive cells was significantly decreased on day 14 compared to day 7 for the controls (*p* < 0.01), the RF-C_3_F_8_ and the MW-C_3_F_8_ implants (*p* < 0.05 for both). In contrast, on day 56 the NK cell count was significant increased for all four implants (*p* < 0.05–*p* < 0.01) compared to day 14. On experimental day 7, the median count for all implants varied around 29 to 37 cells per mm^2^ (IQR 10–61 cells). On day 14 the median significantly decreased for all implants and fluctuated around four to five cells per mm^2^ (IQR 3–8 cells). On day 56 the median ranged from 25 to 41 cells per mm^2^ (IQR 21–53 cells). Compared to the controls a significantly stronger reaction was observed only for MW-C_6_F_6_ on day 14, whereas the number of cells between different implants did not differ on experimental days 7 and 56.

## 4. Discussion

Previous studies have shown that the characteristics of metallic surface microtopography such as roughness, micro-discontinuities, hydrophilicity/hydrophobicity, chemistry and charge have a pronounced influence on the initial attachment process to temporary fracture fixation devices [7,8,9]. In prior studies, it has been demonstrated in vitro [11] and in vivo [12] that plasma technology, precursor chemistry and process parameters influence the cellular response to the modified surface.

The aim of the present study was an in vivo evaluation of different anti-adhesive plasma-fluorocarbon-polymer (PFP) films on titanium alloy samples, investigating their influence on the local inflammatory tissue response after implantation in Lewis rats. For this purpose, Finke et al. developed an anti-adhesive coating for temporary bone fixation devices, using different PFP thin film deposition techniques, microwave (MW: higher electron densities and lower electron energies) or radiofrequency (RF: ion bombardments on growing film) discharge plasma with octafluoropropane (C_3_F_8_) or hexafluorohexane (C_6_F_6_) as precursors. Through both applied processes, surfaces with a very smooth, abrasion resistant, and stable hydrophobic character were produced, designated as MW-C_3_F_8_, MW-C_6_F_6_ and RF-C_3_F_8_ [27]. The physico-chemical differences between the two different coating strategies are characterized in detail by Finke et al. [27]. The X-ray photoelectron spectroscopy (XLS) analysis showed that the F/C ratio for the optimized PFP-films was between 1.5 (PFP-MW) and 1.3 (PFP-RF). Fourier transform infrared reflection absorption spectroscopy (FT-IRRAS) revealed similar spectra for PEP-MW and RF surfaces indicating no major differences between the samples, except for a higher CF2 peak for the PFP-RF samples. AFM measurements showed that the arithmetic roughness Ra for PEP-MW was 4.4nm and for PEP-RF only 3.2 nm [27].

The data presented herein suggest that the type of plasma process technology (microwave vs. radiofrequency) significantly influences the inflammatory reactions. In particular, PFP film samples prepared by radiofrequency discharge plasma (RF-C_3_F_8_) were comparable to controls in vivo and showed a similar low inflammatory response. Additionally, previous in vitro studies conducted by Finke et al. have shown a more efficient inhibition of cellular occupations for hydrophobic RF-C_3_F_8_ films [27]. This effect of hydrophobicity on cell attachment processes could also be observed by Kuhn et al. for fibroblast adhesion, demonstrating that in vitro plasma modified titanium surfaces with activated organo-silicon monomer hexamethyldisiloxane (ppHMDSO+O_2_) led to a diminished colonization and proliferation [16]. The reduced roughness in RF films (3.2 nm) could also contribute to the observed low immune response and would favor this plasma process for short term implantation due to a lower degree of adhesion. Additionally, XPS analyses detected no nitrogen but a trend of increased levels of oxygen in the PFP-RF samples. If significantly increased, imbedded oxygen might possibly lead to oxidative stress further reducing ingrowth.

In accordance with our hypothesis of decreased inflammatory local reactions due to anti-adhesive coatings, the pro-inflammatory M1-like macrophages decreased significantly throughout the study period after an initial increase. This likely represents a switch from acute inflammation to an anti-inflammatory environment around day 7 for all samples, possibly being downregulated by the increasing number of T_reg_ lymphocytes. Surprisingly we did not observe significant changes over time, but rather a relatively constant level of anti-inflammatory M2 macrophages and total T lymphocytes in the peri-implant tissue for the entire study period. Additionally, in accordance with those steady numbers of M2 macrophages, there was, except the temporary increase on day 14 for the MW-C_6_F_6_ implants, overall, no significant change in the number of mast cells. Though it was observed in other studies [21] that mast cells would have been likely to induce a phenotype switch through IL-4 degranulation we were unable to support their findings.

Interestingly the implants discharged in a microwave manner also followed the common pattern of constant cell reduction but when compared to controls presented a significantly higher number of pro-inflammatory CD68+ cells indicating a moderately stronger inflammatory surrounding.

In contrast to those findings, other studies indicate a direct correlation between an activation of the host immune system and the hydrophobicity of metallic alloys [6,29]. Seong and Matzinger hypothesise that exposed hydrophobic structures trigger a DAMP activated immune response [19]. This could be a possible explanation regarding the observed stronger immune reaction evoked by PFP-coated titanium alloys compared to controls. In comparison to the experiments conducted by Moyano et al., the experiments are similar in that both used metals exhibit a hydrophobic surface. However, whereas Moyano et al. injected intravenously hydrophobic nanoparticles, leading to a systemic immune reaction in a mouse model [29], the present study observed a local reaction in peri-implant tissue in a rat model offering significantly fewer contact points for immune cells. In addition, intramuscular implantation resembles the actual clinical situation of long- and short-term implantation devices concerning the inflammatory reaction more closely. While the clinical purpose of fracture-fixation devices lies in bone healing, each bone is typically surrounded by muscles, which are a very well perfused and therefore an especially convenient tissue to survey local inflammatory reactions [12]. With regard to the latter, we recently described an increased amount of NK cells in the peri-implant tissue of hydrophilic positively charged titanium surfaces coated with plasma polymerised ethylendiamine (PPEDA) compared to controls during the acute phase (d7) of inflammation [30]. It is therefore conceivable that the increased adhesion due to hydrophilic surfaces elicits an increased inflammation. Additional data from a previous study supports a surface-dependent expression of cytokines in the serum of Lewis rats with different coated titanium implants [31]. While titanium discs coated with plasma polymerised allyl amine (positive charged, hydrophilic) induced a significant increase in pro-inflammatory cytokines IL-2 and IFN-γ, titanium discs coated with plasma polymerised acrylic acid (negative charged, hydrophobic) expressed steady and lower serum levels of IL-2 and a significant increase in anti-inflammatory IL-4 [31].

M1 macrophages can differentiate into M2 macrophages, known as anti-inflammatory cells, through the release of IL-4 and IL-10 by other modulatory cells [20]. Among those, mast cells are able to recruit and initiate fusion of macrophages into foreign body giant cells (FBGCs) by degranulation of histamine and secretion of IL-4 [21,32]. It should also be taken into consideration that in the present study CD68-positive stained cells were designated as pro-inflammatory M1-like cells and CD163-positive cells were designated as anti-inflammatory M2 macrophages. Until the present day, it is not possible to clearly differentiate histologically between M1 and M2 macrophages in rat tissue using only two markers, since there is no clear marker that is exclusively expressed on only one of the macrophage phenotypes [33,34,35].

The data herein indicate that titanium alloy implants with a radiofrequency discharge plasma (RF-C_3_F_8_) coating had a favorable low short- and long-term immune response in vivo in comparison to discs that were coated in a microwave process (MW). The most pronounced of these observed differences between RF- and MW-processed implants were seen for MHC class II antigen-presenting cells. On day 14 both MW-implant series, MW-C_3_F_8_ and MW-C_6_F_6_, presented a significant increase in stained cells probably representing a much stronger activation of the adaptive humoral response. In contrast, controls and RF-discs showed a significant reduction in MHC class II antigen-presenting cells throughout the study period associated with less severe or non-activation of the adaptive immune system. This change from acute (d7) to intermediate (d14) to chronic inflammation (d56) and the more severe T cell-mediated response was also observed in our total T lymphocytes staining for MW-implants, although only as a tendency.

Within the microwave coated implants the influence of two distinct precursors, C_3_F_8_ and C_6_F_6_, on the inflammatory reaction was biologically examined. Significant differences in the induced local immune reaction could be observed between those two precursors, indicating a more favorable interaction for MW-C_3_F_8_ compared to MW-C_6_F_6_ implants. The most pronounced alteration was observed for mast cells on day 14, when the number of mast cells in the surrounding peri-implant tissue of MW-C_6_F_6_ discs was significantly elevated compared to controls.

Nestin, essential in the induction of myogenic differentiation and regeneration processes was observed to initially increase its expression number with a subsequent significant reduction in the peri-implant tissue until the end of the study period. This is in agreement with several other studies [25,36] highlighting that the process of myogenic muscle regeneration and healing mainly occurs parallel to the acute inflammation response.

Considering the initial assumption that a change from a pro- to an anti-inflammatory environment happened, IL-2R+ regulatory T lymphocytes (T_reg_) are important cells inducing anti-inflammatory environments. All four implants showed a significant increase in T_reg_ cells until day 56 indicating a counter-inflammatory regulation happening at the end of the study period. Prabhakara et al. confirmed in in vitro studies that an early domination of T_reg_ cells could prevent a development of chronic inflammation [37]. In the present study CD25+/IL-2-positive stained cells were designated as T_reg_ cells. The IL-2 receptor complex is not only expressed by T_reg_ but also by CD8+ cells. It consists of three subunits IL-2Rα(CD25), IL-2Rß(CD122), and IL-2R.γ(CD132) [38]. However, the expression pattern of these subunits differs between IL-2-regulated cells, making it possible to histologically separate those inversely acting cells. While CD8+ T cells mainly carry the dimeric IL-2Rß(CD122), and IL-2R.γ(CD132) receptors, regulatory T cells express a high level of trimeric IL-2Rα(CD25) receptors [39,40]. This increase in T_reg_ cells could not be observed in the total amount of T lymphocytes since the number of positively stained cells per mm^2^ was much lower, with a median ranging from 0.95 to 15.75 cells per mm^2^ compared to T lymphocytes with median varying from 23.2 to 34.8 cells per mm^2^ for the four implants investigated in this study.

As expected, natural killer cells, since being part of the innate immune system, were observed to be present in the peri-implant tissue in the acute phase of inflammation with a pronounced decrease until day 14. However, in the time that would likely represent a chronic state of inflammation, a subsequent increase for all samples was observed concurrently with a T_reg_ increase. It is therefore conceivable that NK cells could play a so-far-underestimated role in regulation of adaptive immune cells to a more favorable anti-inflammatory environment preventing excessive immune responses. Supporting this hypothesis, Lu et al. found that NK cells are able to influence lymphocyte expansion in particular and promote the development of CD4+ T-cells, while a blocked interaction resulted in lysis of activated autoreactive T cells [41].

Overall, especially the data for T lymphocytes and antigen-presenting cells indicate that humoral immunological reactions against the anti-adhesive films are likely involved in the biological response and should be therefore investigated in further studies. The anti-adhesive effectiveness should be studied in more detail regarding the relationship between physico-chemical properties and biological response.

## 5. Conclusions

The aim of this study was an in vivo evaluation of different anti-adhesive plasma-fluorocarbon-polymer (PFP) films on titanium alloy samples, investigating their influence on the local inflammatory tissue response after simultaneous implantation in Lewis rats. A differentiated morphometric evaluation of the inflammatory reaction over time was conducted by immunohistochemical staining of cryosections of surrounding peri-implant tissue. Samples were taken on day 7 representing the acute phase of inflammation, on day 14 for an intermediate phase and on day 56 representing chronic inflammation.

In summary, the results of this study support the conclusion that the type of plasma process technology (microwave vs. radiofrequency) significantly influenced the inflammatory reactions. In particular, titanium alloy implants with a radiofrequency discharge plasma coating (RF-C_3_F_8_) had a more favorable low short- and long-term immune response in vivo as well as a more efficient inhibition of osteoblast occupations in vitro. Within the microwave processed discs, implants coated with the C_3_F_8_-precursor had a more favorable elicited cell interaction compared to implants coated with a C_6_F_6_-precursor. Our data strongly support the thesis of decreased inflammatory local reactions due to anti-adhesive coating. This was observed by the significant decrease in pro-inflammatory M1-like macrophages throughout the study period after an initial expected increase, possibly being downregulated by the significant increasing number of Treg-lymphocytes and activated NK cells. Therefore, the present study further supports the potential of low-temperature radiofrequency discharge plasma for temporary implant devices with PFP-coated anti-adhesive surfaces.

## Figures and Tables

**Table 1:** Plasma-fluorocarbon-polymer (PFP) film implants *p*-values; Concerning each marker ED1 (CD68+ monocytes/macrophages), ED2 (CD163+ macrophages), OX6 (MHC class II positive antigen-presenting cells), R73 (T lymphocytes), OX39 (CD25+ (IL-2R+) regulatory T lymphocytes), nestin-positive cells, toluidine blue (mast cells) and ANK61 (NK cells) for comparison of measured percentages of positively stained area or positive cells per mm^2^ in the peri-implant tissue of implant samples, the non-parametric Kruskal–Wallis test with Gaussian approximation was carried out.

Marker	Control	RF C_3_F_8_	MW C_3_F_8_	MW C_6_F_6_
	d 7, 14, 56	d 7, 14, 56	d 7, 14, 56	d 7, 14, 56
ED1	0.035	0.0009	0.0399	0.0236
ED2	0.1163	0.1251	0.636	0.3627
OX6	0.0054	0.0193	0.0014	0.0259
R73	0.0912	0.3203	0.6114	0.4744
Nestin	0.0048	0.0854	0.0003	0.0196
OX39	0.011	0.0007	0.001	0.0057
Toluidine blue	0.4838	0.0949	0.2947	0.0029
ANK61	0.0007	0.0013	0.0034	0.0216

## Data Availability

The data presented in this study are available on request from the corresponding author.
